# Vagus nerve stimulation therapy in people with drug-resistant epilepsy (CORE-VNS): rationale and design of a real-world post-market comprehensive outcomes registry

**DOI:** 10.1136/bmjno-2021-000218

**Published:** 2021-12-23

**Authors:** Arjune Sen, Ryan Verner, James P Valeriano, Ricky Lee, Muhammad Zafar, Rhys Thomas, Katarzyna Kotulska, Ellen Jespers, Maxine Dibué, Patrick Kwan, The CORE-VNS Registry Group, Kate Riney

**Affiliations:** 1 Nuffield Department of Clinical Neurosciences, John Radcliffe Hospital, Oxford, Oxfordshire, UK; 2 Clinical and Medical Affairs, LivaNova Plc, London, UK; 3 Allegheny General Hospital, Pittsburgh, Pennsylvania, USA; 4 Department of Neurology, Ascension Medical Group, Wichita, Kansas, USA; 5 Department of Pediatrics, Duke University School of Medicine, Durham, North Carolina, USA; 6 Translational and Clinical Research Institute, Newcastle University, Newcastle upon Tyne, Tyne and Wear, UK; 7 Royal Victoria Infirmary, Newcastle upon Tyne, UK; 8 Department of Neurology and Epileptology, Children's Memorial Health Institute, Warszawa, Poland; 9 Department of Neurosurgery, Friedrich Schiller University Jena, Jena, Thüringen, Germany; 10 Department of Neuroscience, Central Clinical School, Monash University, Clayton, Victoria, Australia; 11 Epilepsy Unit, Brain Program, Alfred Hospital, Melbourne, Victoria, Australia; 12 Departments of Medicine and Neurology, The Royal Melbourne Hospital, Melbourne, Victoria, Australia

**Keywords:** epilepsy, epilepsy, surgery, paediatric neurology, paediatric neurosurgery

## Abstract

**Introduction:**

The Vagus Nerve Stimulation Therapy System (VNS Therapy) is an adjunctive neuromodulatory therapy that can be efficacious in reducing the frequency and severity of seizures in people with drug-resistant epilepsy (DRE). CORE-VNS aims to examine the long-term safety and clinical outcomes of VNS in people with DRE.

**Methods and analysis:**

The CORE-VNS study is an international, multicentre, prospective, observational, all-comers, post-market registry. People with DRE receiving VNS Therapy for the first time as well as people being reimplanted with VNS Therapy are eligible. Participants have a baseline visit (prior to device implant). They will be followed for a minimum of 36 months and a maximum of 60 months after implant. Analysis endpoints include seizure frequency (average number of events per month), seizure severity (individual-rated categorical outcome including very mild, mild, moderate, severe or very severe) as well as non-seizure outcomes such as adverse events, use of antiseizure medications, use of other non-pharmacological therapies, quality of life, validated measures of quality of sleep (Pittsburgh Sleep Quality Index or Children’s Sleep Habit Questionnaire) and healthcare resource utilisation. While the CORE-VNS registry was not expressly designed to test hypotheses, subgroup analyses and exploratory analysis that require hypothesis testing will be conducted across propensity score matched treatment groups, where possible based on sampling.

**Ethics and dissemination:**

The CORE-VNS registry has already enrolled 823 participants from 61 centres across 15 countries. Once complete, CORE-VNS will represent one of the largest real-world clinical data sets to allow a more comprehensive understanding of the management of DRE with adjunctive VNS. Manuscripts derived from this database will shed important new light on the characteristics of people receiving VNS Therapy; the practical use of VNS across different countries, and factors influencing long-term response.

**Trail registration number:**

NCT03529045.

**Summary**

- CORE-VNS is a global, prospective registry designed to examine the safety and effectiveness of Vagus Nerve Stimulation Therapy System (VNS Therapy) in a real-world setting.
- This registry captures a wide array of clinical outcome measures to assess the broad scope and impact of VNS Therapy.
- Herein, prospective analyses are proposed and registered for study using CORE-VNS data, including details regarding planned subpopulation analyses.

## Introduction

Epilepsy is one of the most common serious neurological conditions worldwide, affecting approximately 50 million people and associated with multiple comorbidities and marked psychosocial impact.^1^ About 35% of people living with epilepsy continue to experience seizures despite taking antiseizure medications (ASMs).^2^ Failure to control seizures has serious repercussions that include a higher risk of depression and suicidal mortality, sudden cardiac death, sudden unexpected death in epilepsy, status epilepticus and deaths due to accidents or injuries.^3–6^ Failure to respond to adequate doses of two ASMs constitutes drug-resistant epilepsy (DRE) that merits consideration of non-pharmacological therapies such as surgical resection, dietary therapy and neuromodulatory interventions.^7^

Of the neuromodulatory therapies for epilepsy, the Vagus Nerve Stimulation (VNS) Therapy System is the most widely available. VNS Therapy was approved for use in people with focal and generalised onset seizures in people of all ages in Europe in 1994; for focal-onset seizures in people over the age of 12 the USA in 1997, and is now used across the globe. Since approval, the technology behind the VNS Therapy system has evolved, and indications for use in, for example, the USA have expanded to include applications in children as young as 4 years.

Early advances in VNS Therapy focused largely on improvements in battery life, device size and usability. In recent models, such as AspireSR (Model 106) and SenTiva (Model 1000), additional functionality is available to assist healthcare professionals and improve clinical outcomes. The AutoStim feature was introduced in the AspireSR device to detect and respond to ictal tachycardia, as an indirect biomarker of seizures, and thereby deliver additional stimulation acutely at the time of a seizure to contain seizure propagation in the brain and/or terminate seizures.^8–11^ The AutoStim feature has since been widely adopted, and VNS Therapy that uses the AutoStim feature (AspireSR and SenTiva) is collectively described as responsive VNS. Additionally, the SenTiva system introduced automatic titration using a predefined protocol (scheduled programming) without the need for clinical visits; the ability to deliver separate output parameters for different time periods in a given day (day/night programming); and access to events and trends data to help in patient monitoring (event monitoring) (table 1).

**Table 1:** VNS systems and features

VNS system	AutoStim responsive VNS Therapy	Scheduled Programming	Day/Night Programming	Event Monitoring
Demi-Pulse	–	–	–	–
AspireSR	+	–	–	–
SenTiva	+	+	+	+

VNS, vagus nerve stimulation.

Of these new features, responsive VNS has been the most extensively studied, both in manufacturer-sponsored approval trials as well as in a series of independent prospective and retrospective single-centre cohort studies.^8–19^ These initial investigations suggest that responsive VNS Therapy offers incremental benefit over traditional, non-responsive VNS and may be associated with an earlier onset of effect.

The purpose of CORE-VNS is to assess the broader real-world use of various features of VNS Therapy and their impact. Here we describe the design of the registry and explore some of the hypotheses that we may be able to test.

## Methods and analysis

The CORE-VNS Post-Market Registry aims to collect data on the ‘real life’ global experience of clinical outcomes and safety for people with DRE treated with adjunctive VNS. This registry represents an opportunity to collect a broad set of endpoints, both clinical and health economic, on a large and diverse patient population enabling substantial subpopulation analysis. This global perspective should provide evidence to guide physicians and their patients, regulators and payers/commissioners on the use of VNS for the management of DRE in multiple global regions.

### Participant eligibility and enrolment

Participants considered eligible for enrolment in CORE-VNS must meet the following criteria:

1. Clinical diagnosis of DRE to be treated with VNS Therapy. People receiving either initial implants or replacement implants are eligible for enrolment.
2. Able and willing to comply with the frequency of protocol-specified visits.
3. Able to voluntarily provide informed consent, by participant or their legal guardian in accordance with institutional policies. In some cases, individuals who are less than 18 years of age may be required to sign an assent affirming their agreement to participate.

There are no specific exclusion criteria for this all-comers registry. Further, local regulatory approvals may limit access to specific VNS Therapy devices for some patient populations.

Participants are considered enrolled on completion of the baseline visit (which includes affirmation of informed consent) and successful implantation of the VNS Therapy system.

There will be no deviation from standard clinical care and only those people who the clinical team decides are suitable for VNS implantation/replacement will be offered the choice to participate. There will be no change to clinical care based on whether an individual chooses to participate or not. Participants may elect to exit the registry at any time without the need to give a reason and with no adverse impact on their clinical care. Participants who are discontinued by the investigators will have reasons documented for discontinuation (eg, early termination or lost to follow-up). Data from participants who withdraw or are withdrawn from the registry will be included to the point of withdrawal.

### Clinical sites

**Figure 1:**
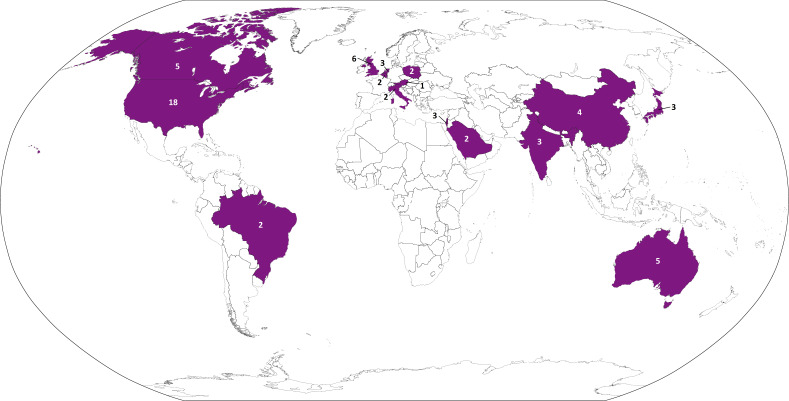
CORE-VNS is a global drug-resistant epilepsy registry that includes sites in Australia, Austria, Belgium, Brazil, Canada, China, India, Israel, Italy, Japan, Netherlands, Poland, Saudi Arabia, UK and the USA. The number listed on or near each highlighted country indicates the number of CORE-VNS sites in that country. A full list of centres and principal investigators can be found in online supplemental appendix A. Note: This map has been adapted from The World Factbook. VNS, vagus nerve stimulation.

The CORE-VNS registry has currently activated 61 clinical sites across 15 countries (figure 1). The sites and investigators supporting this registry are listed in online supplemental appendix A.

10.1136/bmjno-2021-000218.supp1 Supplementary data

### Clinical endpoints

Beyond information on demographics, medical history and genetics (where applicable) in the global DRE population, the registry aims to gather a broad range of clinical outcome measures to assess the purported benefits of VNS. The clinical endpoints of the registry include data collection at baseline as well as 3, 6, 12, 24 and 36 months after implant. Long-term follow-up data may continue annually through 60 months after implant where applicable (table 2).

**Table 2:** Protocol-specified visit schedule and assessments for participants in the CORE-VNS registry. After the first year, patients will return to the clinical site for annual visits, where all endpoint measures will be assessed. At unscheduled visits, only VNS parameter settings, concomitant treatment for epilepsy and safety data will be collected

Target follow-up	Baseline	Implant	Follow-up (post implant)					
	−45 to 5 days before implant	0	Titration visit(s)	3 months (±45 days)	6 months (±45 days)	12/24/36 months (±45 days)	Long-term follow-up *Every 12 months until 60 months or study end (±60 days*)*	Unscheduled visits
Informed consent/assent/data privacy	X							
Inclusion/exclusion criteria	X							
Demographics and medical history	X							
VNS Therapy implant/revision/explant		X	*As needed*	*As needed*	*As needed*	*As needed*	*As needed*	*As needed*
VNS Therapy parameter settings		X	X	X	X	X	X	X
Concomitant treatments for epilepsy†	X			X	X	X	X	
Rescue medication use	X			X	X	X	X	
Seizure frequency	X			X	X	X	X	
Maximum seizure-free period	X			X	X	X	X	
Seizure severity/postictal severity	X			X	X	X	X	
Quality of life	X			X	X	X	X	
Quality of sleep (PSQI or CSHQ)‡	X			X	X	X	X	
Healthcare utilisation	X			X	X	X	X	
VNS Therapy feature use (if applicable)			X	X	X	X	X	X
Deaths and AEs related to VNS (if applicable)§		X	X	X	X	X	X	X
Pregnancy reporting form (if applicable)	*As needed*	*As needed*	*As needed*	*As needed*	*As needed*	*As needed*	*As needed*	*As needed*
Device deficiencies (if applicable)		X	X	X	X	X	X	X
Study completion or withdrawal			*At study exit*	*At study exit*	*At study exit*	*At study exit*	*At study exit*	*At study exit*

*During the long-term follow-up phase, subjects will have yearly follow-up visits until 60 months post implant or until study end (whichever comes first).

†Pharmacological and non-pharmacological.

‡PSQI: Pittsburgh Sleep Quality Index for subjects ≥18 years of age at baseline/CSHQ: Children Sleep Habit Questionnaire for subjects 2–17 years of age at baseline.

§AEs possibly, probably or definitely related to VNS Therapy per investigator assessment (including those with unknown relationship).

AEs, adverse events; CSHQ, Children Sleep Habit Questionnaire; PSQI, Pittsburgh Sleep Quality Index; VNS, vagus nerve stimulation.

Clinical assessments include: average seizure frequency per month for the 3 months prior to each protocol-specified visit and maximum seizure-free period over 90 days prior to each protocol-specified visit. Seizure severity and postictal severity are based on participant-rated categorical outcome (very mild; mild; moderate; severe; and very severe). Quality of life assessment is based on a participant-rated categorical outcome (very good: could hardly be better; pretty good; good and bad parts about equal; pretty bad; or very bad: could hardly be worse). Given the anticipated recruitment and the duration of the study, Likert scales were selected to limit the impact of visit duration on enrolment and participant retention.

On account of previously reported interactions between VNS and sleep apnoea and the limited evidence in this area,^20–22^ quality of sleep assessment was collected through validated scales. Participants who are over 18 years of age at baseline are requested to complete the Pittsburgh Sleep Quality Index (PSQI) at each protocol-specified visit, including baseline.^23^ Participants who are between 2 and 18 years of age at baseline complete the Children’s Sleep Habit Questionnaire.^24–26^ Participants who are less than 2 years of age at baseline are not assessed for sleep quality.

### Use of ASMs, other therapies and rescue drugs

The type and dosage of all concomitant ASMs are recorded at each protocol-specified visit. Other therapies such as ketogenic diet are also recorded. During the baseline visit, the number of times a rescue drug had been used over the past 12 months is captured, as is rescue drug use between each follow-up visit.

### Healthcare resource utilisation

The number of seizure-related emergency department (ED) visits and seizure-related hospitalisations, as well as the duration of those stays, that have occurred since the previous visit are collected at each visit. At the baseline visit, these outcomes are collected through the previous 12 months. Clinical visits related to VNS programming are also collected.

### VNS Therapy feature use and rationale

Use of optional VNS Therapy features, such as magnet mode, AutoStim, scheduled programming and day/night programming are collected, where applicable, at each visit. In people with Model 1000 (SenTiva) generators, the clinician’s reasons for use of these features are also recorded.

### Safety endpoints

The safety endpoints include all deaths, regardless of cause and relationship to the device or procedure; adverse events related to VNS (including any device deficiencies); and device deficiencies that do not lead to adverse events such as high lead impedance or inaccurate device information.

Adverse events are assessed as ‘possibly’, ‘probably’ or ‘definitely’ related to VNS exclusively by the investigators. Investigators could also specify that they did not know if there was a relationship between the adverse event and the VNS device. No predetermined relationships between adverse events and the treatment were provided to investigators who were expected to make a pragmatic classification of likelihood based on clinical data. All sites were diligently monitored for adverse event reporting and any discrepancy between the clinical record and the study capture forms were highlighted and explanations for this discrepancy documented.

Adverse events are recorded from time of implant until exit from the registry and will be summarised as part of the safety results. All adverse events are also reported to the sponsor’s safety specialists for assessment of their relation to the VNS device for purposes of regulatory reporting requirements. If the registry sponsor and the investigator disagree on the source of an adverse event, both the sponsor’s and investigator’s assessments will be recorded in the database, and the investigator’s assessment will be used for analysis.

### Statistical analysis

Given the exploratory nature of the data set and the principal aim to provide preliminary information on clinical outcome and safety data, the sample size is not calculated based on a statistical power. There are no prospective requirements for enrolment at each centre, meaning that certain centres may contribute a much larger percentage of the study population than others.

Three analysis populations are prospectively defined: modified enrolled population (mENR), modified safety population (mSAF) and the full analysis set (FAS). The mENR or ‘screened’ population are participants with a signed and dated informed consent who meet all registry requirements, consistent with ISO-14155:2011. The mSAF population is defined as all participants in the mENR population who underwent an implantation procedure with the VNS Therapy system, whether that procedure was successful or not. The FAS population includes all participants in the mSAF population with at least one post-baseline efficacy (clinical or performance) assessment. The FAS will be used for efficacy and performance summaries, including all subpopulation analyses. All safety analyses and subject disposition summary will be assessed using the mSAF population.

Further subpopulation analysis will occur in the following groups, provided they include more than 1% of the total subject count:

- Age class (paediatric <18 years of age or adult ≥18 years of age).
- Implant type (new implant or replacement implant).
- Implant timing (Early: ≤4 ASMs failed or <5 years since initial epilepsy diagnosis at time of implant or Late: All other patients).
- Seizure type/focus (generalised, focal, combined or unknown).
- Specific epilepsy syndrome (if known).
- Epilepsy aetiology (if known).
- VNS Therapy system model.
  ­ Non-responsive VNS Therapy device: Pulse (Model 102), Pulse Duo (Model 102R), Demi-Pulse (Model 103), Demi-Pulse Duo (Model 104), AspireHC (Model 105).
  ­ AspireSR (Model 106).
  ­ SenTiva (Model 1000).
- Feature use (AutoStim ON vs OFF; day/night ON vs OFF; scheduled programming ON vs OFF).

Principal analysis of the full cohort data will occur at 3 years of follow-up and will be summarised using descriptive statistics for the relevant analysis population (mSAF or FAS). Intermediate analysis will also be conducted for the full cohort at each year after implant, and topics of this analysis are further described in the following section. Continuous variables will be summarised using the number of observations, mean, median, SD, minimum and maximum values. Categorical variables will be summarised using the number of observations and percentages. Absolute scores and changes from baseline with 95% CIs will be reported for each clinical endpoint at each follow-up visit. VNS-related adverse events, including serious adverse events and deaths, will be presented by system organ class and preferred term as total counts and percentages. These adverse events will be further described by their relationship to VNS implant and/or stimulation and the current status (eg, ongoing, resolved, death, chronic or unknown).

### Ethics and dissemination

The registry is being conducted in accordance with the ethical principles that have their origin in the Declaration of Helsinki, and that are consistent with Good Clinical Practice described in ISO 14155, and the applicable regulatory requirement(s) of each participating clinical centre. Any additional requirements imposed on the study by a clinical centre’s Institutional Review Board or Ethics Committee will be followed.

Beyond the principal analysis of these results described in the previous section, which will focus on our assessment of the primary clinical and safety outcomes in the FAS and mSAF populations, additional exploratory analyses are expected within this database. The CORE-VNS registry will collect a variety of exploratory clinical outcomes that have not been robustly characterised in a large population treated with VNS. These exploratory analyses will constitute a significant portion of the dissemination strategy for this large database. All results will be submitted to peer-reviewed scientific journals and may also presented as abstracts at large epilepsy congresses with international audiences.

For some of the following analyses, descriptive statistics will be sufficient to describe how practice and outcomes vary, but in other cases direct hypothesis testing is warranted. If hypothesis testing is required for these exploratory analyses, propensity scoring based on baseline demographics and clinical histories will be employed to create matched treatment groups for comparison.

### General demographics and medical history

The CORE-VNS registry is designed to be the largest, prospective, international data set ever collected of people receiving VNS for DRE. This creates an opportunity to assess the global demographic profile of people who receive adjunctive VNS. Regional variances in age, sex, ethnicity, race, aetiology (structural, genetic, infectious, metabolic, immune, unknown), epilepsy syndrome (childhood absence, juvenile absence, juvenile myoclonic, infantile spasms, West, Dravet, Lennox-Gastaut, tuberous sclerosis, continuous spikes/slow waves during sleep, electrical status epilepticus, epilepsia partialis continua, unknown, other), epilepsy type (focal, generalised, combined, unknown), history of brain surgery, family history of seizures, comorbidities, duration since epilepsy diagnosis and medical history of people who proceed to VNS will be investigated. Furthermore, we aim to better understand the prescribing history of failed ASMs in the DRE population.

### Use of VNS in genetic epilepsies

At some institutions participating in the CORE-VNS registry, genetic profiling of patients with DRE is included as part of their medical assessment. In clinical application, VNS has often been applied in cases of putative genetic epilepsies, for example, Dravet syndrome, some cases of Lennox-Gastaut syndrome and other epileptic encephalopathies, even if the therapy has not been directly investigated within these populations in randomised clinical trials. We will capture the regional practices of genetic testing in DRE as well as exploring the frequency of use of VNS in those with confirmed/suspected genetic epilepsy. We will examine the effectiveness of VNS on seizure and non-seizure outcomes in these patients. Due to the wide range of genetic mutations contributing to epilepsy and as the registry is open to all-comers, it is impractical to a priori define subpopulations for this analysis.

### Regional practice and feature use

This registry aims to better understand regional differences in the implementation of VNS. Specifically, there is interest in identifying regional variances in clinical and safety outcomes associated with different implementations of VNS dosing and standard clinical practice. Newer models of VNS devices have only recently become available in some of the regions participating in the registry, so differences may be apparent in the use of new features. The features available in these newer models offer more programming flexibility and the potential for improved clinical and safety outcomes, but may also add complexity to the therapy. In this study, rationale for the selection of a Model 1000 ‘SenTiva’ generator is collected by ranking the relative importance of each feature in the eventual decision. The rationale options for device or feature selection include: scheduled programming, day/night programming, low heart rate detection, prone position detection, events and trends, availability of AutoStim with a smaller generator.

### Titration guidance and scheduled programming

The primary limitations of previous studies of VNS in providing quality guidance on dosing and titration were limited sampling of all device parameter combinations and lack of programming data collected during the titration period.^27–30^ CORE-VNS will collect detailed titration data prospectively. A generalised linear mixed model will be developed to assess the sensitivity of clinical and safety outcomes to specific programming settings. The relationship between time-to-dose and time-to-response will be assessed using a Cox proportional hazard model to characterise the impact of rapid titration (titration in <3 months) on clinical outcomes, as opposed to slow titration (>6 months). Titration period will be defined as time taken to achieve either an optimal programming output as defined by the mixed model, or as time taken to the reach the minimum recommended output current defined in the device manual.

### ASMs and VNS Therapy

In accordance with the approved product label, VNS Therapy is prescribed concomitantly with other therapies. Despite this, little guidance has been offered to suggest whether drug–device combination therapies can enhance efficacy or safety outcomes. Polypharmacy contributes to the current lack of evidence, as it creates significant challenges in creating consistent subpopulations for analysis. The size of the CORE-VNS registry may allow insight whether certain drug classes (eg, benzodiazepines; sodium channel blockers) in combination with VNS are associated with more favourable outcomes. The question of whether VNS reduces average drug burden over time will also be explored.

### Concomitant therapies with VNS Therapy

The CORE-VNS inclusion criteria specify that participants must have pharmacoresistant epilepsy. A recent VNS registry has reported a number of people are concomitantly treated with VNS and another non-pharmacological DRE therapy (such as dietary therapy or other neurostimulation therapies) or have previously undergone epilepsy surgery.^29^ Exploring the possible synergistic benefits of VNS with other non-pharmacological therapies is of interest. Given the global scope of the trial and the limited worldwide access to other device-based therapies, it is anticipated that the potential synergistic effect of VNS and ketogenic diet^31^ will be the primary focus in this context. Deep brain stimulation is approved for use in approximately half of the study centres and responsive neurostimulation is approved for use in approximately one-third of the study centres. These devices are not approved for concomitant use with other neurostimulation technologies, so off-label use with VNS is expected to be very infrequent. Pursuant to the statistical analysis plan, interactions between these therapies and VNS will only be assessed if the number of participants within a given subpopulation exceeds 1% of the total study population and will be conducted in accordance with the statistical techniques defined previously. Any participants that receive another device during the follow-up period will be followed normally.

### Healthcare resource utilisation by people receiving VNS

The DRE population uses a disproportionate amount of the healthcare resources accounted for by people with epilepsy, which is characterised in the skewed nature of epilepsy healthcare cost distributions.^32^ Costly ED visits are significant drivers of the healthcare resource utilisation in this population. We will assess, on a regional basis, how VNS impacts ED utilisation and overall healthcare utilisation. We will also assess whether utilisation of scheduled programming can reduce the number of office visits required to titrate patients to dose.

### VNS and sleep quality

VNS has been reported to exacerbate and/or induce sleep apnoea in multiple independent case series.^20–22^ Apnoeic events are known to decrease quality of sleep and overall health outcomes. At the same time, uncontrolled epilepsy can impact sleep quality and is associated with increased incidence of obstructive sleep apnoea.^33^ Better understanding of the risk–benefit profile of VNS with respect to quality of sleep and health outcomes seems warranted.

VNS parameters have long been understood to play a role in this adverse effect, and VNS-induced apnoea can be managed by reducing the intensity of stimulation.^34^ Day/night programming was developed in part to help ameliorate apnoeic events through changes in programming parameters during sleep, but the implementation of this technology for reducing incidence of VNS-induced apnoea has not been examined. Therefore, whether day/night programming can improve sleep quality will be investigated. In adult patients, this analysis will be limited to participants with sufficient cognitive ability to provide a complete PSQI as the tool is not validated for use by caregivers.

### The global safety profile of VNS

VNS has been established as a well-tolerated and safe therapy. This finding has been replicated in multicentre randomised controlled trials, registries, single-centre experiences and case series. Most of the global research experience with VNS, however, has occurred in only a few countries. CORE-VNS presents an opportunity to better understand the safety profile of VNS in multiple regions where it has not been previously studied, especially those regions with more recent access to the therapy. Also, certain device components (such as the newest leads) or features (such as responsive VNS) have not been assessed in a comparative fashion versus previous models of these components in a real-world setting.

Patients involved in the study can elect to undergo a device explantation, for any reason. The rationale for explantation is recorded in the case report form associated with that study event. The explantation surgery case report form includes rationale for the explant, such as: death, adverse event(s), lack of efficacy, VNS Therapy no longer desired, MRI, battery depleted, battery nearing depletion, unknown, other.

### Design limitations

Although VNS Therapy has been approved for the treatment of DRE for nearly 30 years, systematic evaluation of its use in the real-world setting has been lacking. The clinical benefits of the latest advances in model features have not been well studied. Given the prospective design, international recruitment and scale, CORE-VNS is ideally suited to generate the much needed long-term evidence to inform the optimal use of modern VNS.

Nonetheless, the study has several limitations. The primary limitation in the protocol design is that the registry only explores the effects of VNS in those who receive it. There is no direct comparison with a parallel group who do not receive VNS. Therefore, the incremental benefit of adding the therapy to a treatment paradigm can only be assessed by comparing with the participant’s own baseline.

The registry did not enrol all patients with VNS. While all sequential patients were considered for enrolment, the inclusion criteria for the study required that subjects and caregivers be willing and able to comply with the frequency of study visits, and assent/consent to participate. Because the inclusion criteria were assessed before the creation of any study documentation linked to a potential participant, no study documentation exists to assess the risk of bias from non-included patients, which could potentially impact the generalisability of the results. Finally, this registry is observational and was not specifically designed to address any of the exploratory analyses described here with adequate statistical power. It is expected that the large size of this registry will help to reduce the risk of undersampling.

There are practice-specific differences in implementation of VNS. The manufacturer’s recommendations on titration and programming may not be closely followed at all centres, including some centres and practices included in the CORE-VNS registry. There are many legitimate, person-centred reasons for this interpractice variability (eg, tolerability, lifestyle) and there are also fundamental differences in hospital-led and company-led training between regions (including regions within the same country). These differences in therapy implementation may confound regional assessment of clinical and safety outcomes. In all regional analyses, careful attention will be paid to marked variance in VNS settings (as compared with global averages based on this study and historical VNS registry data).

The design of CORE-VNS allows for a prospective assessment of programmed settings through the titration window. In many people, these settings are collected in electronic records along with other clinical and safety outcomes data. Such records are not collected at titration visits, which do not assess clinical and safety outcomes. To mitigate this, an electronic capture of the programming history from each programming tablet will be used in the registry. These data will be used to develop a clear picture of the titration period from each site and aim to eliminate the risk of missing data fields in titration logs.

Lastly, COVID-19 has impacted this registry both from a recruiting and follow-up perspective. CORE-VNS began in 2018, so until the first quarter of 2020 participants were able to attend follow-up visits in person according to standard programming and titration schedules. During lockdowns imposed during the pandemic, some participants missed titration visits, or these visits were delayed. While participants with newer programming features like scheduled programming may have been less impacted, concern remains regarding the impact of these titration delays on therapeutic efficacy. A subgroup analysis by enrolment year may clarify the extent of this impact.

### Study status

To date, 823 participants have been enrolled in the registry at 61 centres. Enrolment closed by the start of June 2021. Follow-up of participants is expected to continue as planned after termination of enrolment.
