# Supplementary material for: Vagus nerve stimulation therapy in people with drug-resistant epilepsy (CORE-VNS): rationale and design of a real-world post-market comprehensive outcomes registry
Source: BMJ Neurol Open. 2021 Dec 23;3(2):e000218. doi: 10.1136/bmjno-2021-000218 (PMC8705076; doi:10.1136/bmjno-2021-000218)
Supplement: Supplementary data [file bmjno-2021-000218supp001.pdf]

Appendix A. List of Principal Investigators and Institutions Participating in the CORE-VNS Registry

**Name of Institution**	**Name of Principal Investigator**	**Country**
Queensland Children's Hospital	Riney, Kate	Australia
The Royal Children's Hospital	Harvey, Simon	Australia
The Royal Melbourne Hospital	Kwan, Patrick	Australia
The Alfred Hospital	O'Brien, Terry	Australia
Perth Children's Hospital	Nagarajan, Lakshmi	Australia
Kepler Universitätsklinikum Neuromed Campus	von Oertzen, Tim J.	Austria
UCL - Cliniques Universitaires Saint-Luc	El Tahry, Riem	Belgium
UZ Gent	Vonck, Kristl	Belgium
Fundação Felice Rosso, Hospital Felício Rocho	Julião de Oliveira, Andréa	Brazil
Instituto Estadual do Cérebro Paulo Niemeyer (IEC)	D'Andrea Meira, Isabella	Brazil
Centre Hospitalier de l'Université de Montréal	Keezer, Mark	Canada
Montreal Neurological Institute and Hospital	Veilleux, Martin	Canada
McGill University Health Centre, Montreal Children's Hospital	Myers, Kenneth	Canada
London Health Sciences Centre	Burneo, Jorge	Canada
London Victoria Hospital Pediatric Center	Andrade, Andrea	Canada
Sanbo Brain Hospital	Luan, GuoMing	China
Xuanwu Hospital Capital Medical University	Li, Yongjie	China
Guangzhou Women and Children's Medical Center	Li, Fangcheng	China
Renji Hospital Shanghai Jiaotong University School of Medicine	Xu, JiWen	China
St. John's Medical College Hospital	Sarma, Gosala Raja	India
P. D. Hinduja Hospital and Medical Research Centre	Udani, Vrajesh	India
Deenanath Mangeshkar Hospital and Research Centre	Kurwale, Nilesh	India

Name of Institution	Name of Principal Investigator	Country
Sheba Medical Center	Tzadok, Michal	Israel
Tel Aviv Sourasky Medical Center	Fahoum, Firas	Israel
Schneider Children's Medical Center of Israel	Goldberg-Stern, Hadassa	Israel
Ospedale Bellaria	Tinuper, Paolo	Italy
Bambino Gesù Pediatric Hospital	Specchio, Nicola	Italy
NCNP National Center Hospital of Neurology and Psychiatry	Iwasaki, Masaki	Japan
The University of Tokyo Hospital	Shimada, Seijiro	Japan
National Hospital Organization Nagasaki Medical Center	Ono, Tomonori	Japan
Stichting Epilepsie Instellingen Nederland (SEIN)	Gunning, Boudewijn	Netherlands
Kempenhaeghe	Wagner, Louis	Netherlands
Sophia Kinderziekenuis	Neuteboom, Rinze F.	Netherlands
Uniwersyteckie Centrum Kliniczne im. Prof. K. Gibińskiego	Krzystanek, Ewa	Poland
Children’s Memorial Health Institute	Kotulska-Jóźwiak, Katarzyna	Poland
King Faisal Specialist Hospital & Research Center – Jeddah	Al-Said, Youssef	Saudi Arabia
King Faisal Specialist Hospital & Research Center - Riyadh	Alkhateeb, Mashael Omar	Saudi Arabia
Southmead Hospital (North Bristol NHS Trust)	Sieradzan, Kasia	UK
King’s College Hospital	McCormick, David	UK
Royal Victoria Infirmary Hospital	Thomas, Rhys	UK
Norfolk & Norwich University NHS FT	Cochius, Jeffrey	UK
John Radcliffe Hospital, Oxford	Sen, Arjune	UK
Royal Hallamshire Hospital	Reuber, Markus	UK
Child Neurology Consultants of Austin	Keough, Karen	USA
Dent Neurosciences Research Center, Inc.	Frost, Marc	USA
St. Mary's Hospital & Medical Center	Collier, Marie	USA

Name of Institution	Name of Principal Investigator	Country
Hawaii Pacific Neuroscience	Liow, Kore	USA
Via Christi Health	Lee, Ricky	USA
Wake Forest University	Boggs, Jane	USA
Research Institute of Orlando	Sadek, Ahmed	USA
Le Bonheur Children's Hospital	Wheless, James	USA
Columbia St. Marys	Morris, George	USA
University of Pennsylvania	Gelfand, Michael	USA
University of Pittsburgh Medical Center	Bagic, Anto	USA
Allegheny Neurological Associates	Valeriano, James	USA
Duke University School of Medicine	Zafar, Muhammad	USA
University of Texas Health Science Center – San Antonio	Leary, Linda	USA
State University of New York	Zhou, Xiangping	USA
Pediatric Epilepsy and Neurology Specialists	Ferreira, Jose	USA
Georgetown University Medical Center	Motamedi, Gholam	USA
Valley Health System	Lyons, Paul	USA
